# Causal Attributions in Breast Cancer Patients Planning to Undergo Adjuvant Endocrine Therapy

**DOI:** 10.3390/ijerph18115931

**Published:** 2021-05-31

**Authors:** Seul Ki Park, Yul Ha Min, Minsun Lee, Sae Byul Lee

**Affiliations:** 1 Red Cross College of Nursing, Chung-Ang University, Seoul 06974, Korea; gomtty3@cau.ac.kr; 2 College of Nursing, Kangwon National University, Chuncheon-si 24341, Korea; 3 College of Nursing, Seoul National University, Seoul 03080, Korea; minsunlee1989@snu.ac.kr; 4 Division of Breast Surgery, Department of Surgery, University of Ulsan College of Medicine, Asan Medical Center, Seoul 05505, Korea; newstar153@ulsan.ac.kr

**Keywords:** breast neoplasm, causality, perception

## Abstract

The aim of this study was to explore causal attributions among Korean breast cancer patients who were planning to undergo adjuvant endocrine therapy (AET) as well as the relationships between patient demographic and clinical characteristics and their causal attributions. Causal attributions were assessed with an open-ended response item, which asked patients to list what they thought were the three most important causal factors of their illness. The relationships between patient characteristics and causal attributions were determined through univariate analysis, and the relationships between causal attributions were obtained using social network analysis. A total of 299 participants provided 707 responses. Stress, diet, and exercise were believed to be the three most likely causes of breast cancer. There were no significant differences between causal attributions and the age, education level, marital status, or cancer stage of patients. However, there were differences in the associations between personality, genetics, and reproductive history and patient-identified causal attributions according to the patients’ family history of cancer. Patients with a family history of cancer were more likely to believe that personality and genetics/family history were causes of breast cancer compared to patients without such a history. Therefore, it is necessary to educate patients to perceive stress and lifestyle-related factors as modifiable causal factors in order to have a positive effect on their adherence to AET.

## 1. Introduction

Breast cancer is the most common cancer; it is the leading cause of cancer-related death among women in the world [1], and it is the second-highest type of cancer diagnosed in Korean women [2]. More than 23,500 new cases of breast cancer were diagnosed among Korean women in 2018, and that incidence accounts for 20.5% of all cancer in Korean women [2]. However, the 5-year relative survival rate for breast cancer has increased from 79.2% between 1993 and 1995 to 93.2% between 2013 and 2017 [3]. Developments in endocrine therapy, chemotherapy, and target therapy have made a significant contribution to the improvement in breast cancer survival [3]. Especially for women with hormone receptor-positive tumors, clinical trials have shown that the survival rate is improved by the use of adjuvant endocrine therapy (AET) for at least 5 to 10 years [4,5,6]. AET is considered a standard treatment to reduce cancer recurrence [4,5,6]. However, the long-term use of these medications makes patient adherence challenging.

Until now, various factors among women, such as age; genetic factors; family history; estrogen exposure; and lifestyle factors, such as obesity, alcohol consumption, and a lack of physical activity, are known to contribute to breast cancer [7,8,9,10,11]. However, some breast cancer patients perceive factors other than those validated by experts as the cause of breast cancer [12,13], and these factors vary according to patient experience and culture [14]. For example, in a study conducted with Brazilian breast cancer patients, the most common attributions were to psychological factors (47.8%), such as stress and personality [12]. The leading causes perceived by breast cancer patients in China were stress-related factors (17.3%) and psychological factors/personality (17.0%) [15]. The most common causal theme was stress in a qualitative study that explored causal attributions among Chinese American, Korean American, and Mexican American breast cancer survivors [16]. Psychological factors are the most common causal attributions in those studies but are inconsistent as the main causes of breast cancer as validated by the scientific community. These factors are likely to have much smaller roles in carcinogenesis [12,17]. On the other hand, another study in America reported that most breast cancer survivors tended to attribute their illness to lifestyle (60.8%) [17]. Experts highlight the importance of lifestyle factors in controlling and modifying cancer risk [18]. Health beliefs and behaviors are reported to be influenced by culture [16]. In some cultures, cancer is attributed to an external locus of control, which is the belief that cancer depends on external factors [12]. In other cultures, the causes of cancer tend to be perceived as internal and under the individual’s own control [19].

It is known that patient attribution of breast cancer causes affects coping and adjustment to breast cancer treatment and recovery [20]. Causal attributions focused on non-modifiable and uncontrollable risk factors, such as personality, fate, and stress, were associated with a greater fear of cancer recurrence [21] and lower overall quality of life [22]. It is possible that patients feel hopeless because they believe that their cancer was caused by factors that they had no control over or could not change. However, causal attribution focused on modifiable and controllable causes, such as lifestyle-related factors, tends to result in behaviors oriented to coping with the disease. Patients who believed that an unhealthy diet, insufficient exercise, or alcohol consumption contributed to their cancer were more likely to modify their relevant behavior [20]. Internal locus of control promotes medication adherence in breast cancer patients receiving AET [23].

The causal attributions of breast cancer may vary depending on patient characteristics, including age, education level, and family history of cancer. In previous studies that explored the perception of illness associated with patient sociodemographic or clinical characteristics using the Illness Perception Questionnaire (IPQ) or the brief IPQ (BIPQ), which is a representative tool for assessing illness perceptions, higher educated survivors perceived more personal control and had a greater understanding of their disease [14,15]. Patients who had a family history of breast cancer were found to believe that treatment is more helpful to them than those without such a history [14]. Older breast cancer patients attributed their disease to more modifiable factors than younger patients [17]. However, these studies explored overall illness perceptions, and few studies have focused on the relationships between patient causal attributions and patient characteristics. In addition, even in a study that explored patient causal attributions [17], the causal attributions were categorized into two groups: modifiable and non-modifiable, and the relationships between specific causal attributions and patient characteristics were not described.

In this study, we explored causal attributions identified by Korean breast cancer patients planning to undergo AET and the relationships between their causal disease attributions and their demographic and clinical characteristics. The results of this study will allow healthcare providers to deliver more efficient care and education to breast cancer patients.

## 2. Materials and Methods

### 2.1. Design

This was a descriptive correlational design.

### 2.2. Sample and Settings

**Figure 1:**
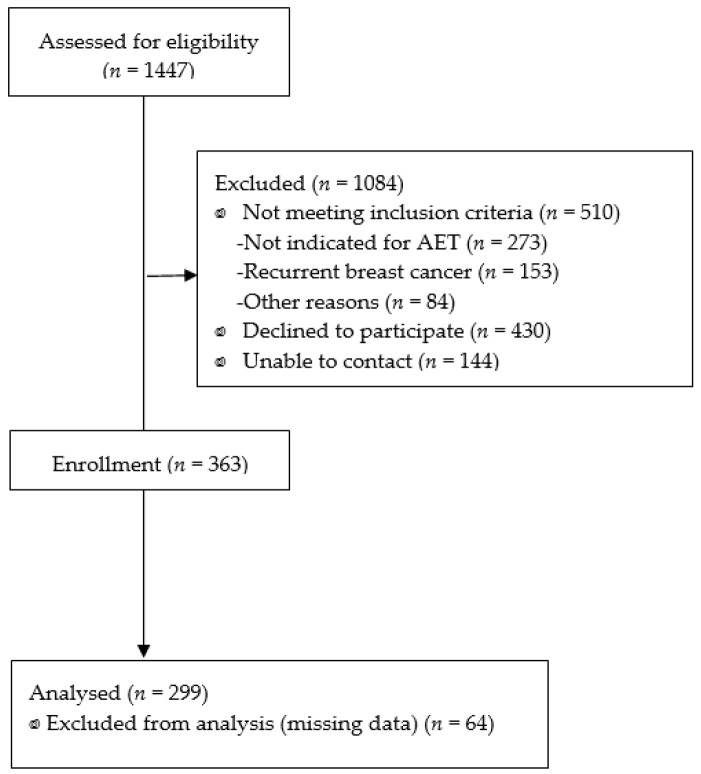
The flow of study participants selection.

Patients were recruited at the Asan Medical Center, a university-affiliated hospital in Seoul, South Korea. Figure 1 shows the flow of participant selection. A total of 1447 consecutive women who were histologically confirmed to have breast cancer at the Asan Medical Center were screened for eligibility from November 2016 to January 2018, at the time they were admitted for surgery. Inclusion criteria were: cases with hormone receptor-positive breast cancer; an age of ≥20 years at diagnosis; and definitive surgery followed by AET irrespective of chemotherapy. Exclusion criteria were local or regional recurrent tumors or a history of psychiatric or neurologic illness. Patients completed a self-report survey including the BIPQ following surgery and before starting AET. Among 937 eligible patients, 510 did not meet the inclusion criteria (273 not indicated for AET, 153 with recurrent breast cancer, and 84 with other reasons) and 574 did not consent to participate in this study (unable to contact 144 patients and 430 declined to participate); thus, 363 patients participated in this study. Data from 64 patients were excluded as a result of missing causal factors in their illness. In total, data from 299 patients were included in this analysis.

### 2.3. Data Collection

The demographic and clinical characteristics of the participants were extracted from electronic medical records with the consent of the patients. Demographic characteristics included age, educational level, marital status, employment status, family history of cancer, and family history of breast cancer. Clinical characteristics included cancer stage, type of surgery, chemotherapy, radiation therapy, and menopausal status.

Causal attribution was assessed with the Korean version of the BIPQ (BIPQ-K) [24], a nine-item scale assessing cognitive and emotional representations of illness, illness comprehensibility, and causal representation. The assessment of the causal representation is completed through an open-ended response item, which asks patients to list the three most important causal factors of their illness. In the BIPQ validation paper [25], the authors stated that, in some cases, it may be best to analyze only the first-ranked cause; whereas, in other cases, it may be better to include all three of the causes indicated by the patients. We include all three of the causes in this study.

### 2.4. Data Analysis

The demographic and clinical characteristics of the patients were analyzed using descriptive analyses. The causal attributions of the patients were analyzed using descriptive analyses and were ranked from the first to the third cause. The research team fully reviewed the responses written by the patients in a free-text format and coded the data based on 6 categories and 23 subcategories that were derived from the literature (Table 1) [12,17,21,22,26]. The data were coded independently by three researchers, and disagreements were resolved by consensus. The associations between demographic and clinical characteristics and causal attributions were analyzed using univariate analysis (χ2 test). We classified the data into two groups according to the family history of cancer. We performed social network analysis to examine and visualize the relationships between causal attributions that appeared together in each answer within the two groups [27]. The nodes in the network represent the causal attributions, while the edges indicate relationships between the nodes. The determination of node activity was based on the degrees corresponding to the number of direct connections to the node. The strength of the relationships between the nodes was based on the edge weights corresponding to the number of interactions [28]. Descriptive and univariate analyses were performed using IBM SPSS Statistics for Windows (version 22.0, Armonk, NY, USA). The network of the relationships between causal attributions was constructed using NetMiner 4.4.3.e (Cyram Inc., Seoul, Korea).

### 2.5. Ethical Considerations

All participants completed written informed consent forms approved by the Institutional Review Board of the Asan Medical Center and Kangwon National University (IRB nos. 2016-0351 and KWNUIRB-2020-11-001-002).

## 3. Results

### 3.1. Characteristics of the Patients

A total of 299 patients were included in the study. The demographic and clinical characteristics of the patients are presented in Table 2. The mean age of the patients was 46.75 (SD = 8.18, range 22–70) years, and most were married (86.0%). Overall, 61.9% of patients had at least a bachelor’s degree and 51.2% were currently employed. A total of 43.5% and 10.7% of the patients reported having a family history of any cancer and breast cancer, respectively. About a quarter of patients (24.4%) were menopausal. Most of the participants had stage I, II, or III (87.0%) cancer and had undergone conservation surgery (80.9%). In the course of their treatment, 33.4% and 82.9% of the participants reported having chemotherapy and radiation therapy, respectively.

### 3.2. Causal Attributions of Breast Cancer

**Figure 2:**
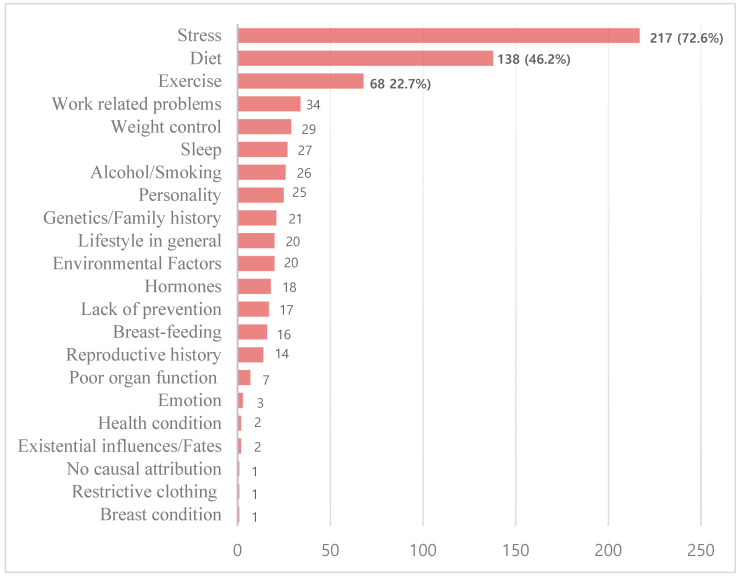
Frequency (number) of causal attribution categories reported by breast cancer patients.

A total of 299 patients provided 707 responses to the open-ended question assessing the three most important causes of breast cancer. Of the total patients, 167 (55.9%), 74 (24.7%), and 58 (19.4%) women wrote three, two, and one answers, respectively. Figure 2 shows the ranking of all responses. Overall, the causal attribution that was mentioned most frequently was stress (217, 72.6% of all patients), followed by diet (138, 46.2%), exercise (68, 22.7%), work-related problems (34, 11.4%), and weight control (29, 9.7%). In the first causal attributions, the most frequently mentioned was stress (147, 49.2%), followed by diet (40, 13.4%), and exercise (15, 5.0%). In the second causal attributions, the most frequently mentioned cause was diet (55, 18.4%), followed by stress (47, 15.7%), and exercise (30, 10.0%). In the third causal attributions, the most frequently mentioned cause was diet (43, 14.4%), followed by exercise (23, 7.7%), and stress (23, 7.7%).

### 3.3. Associations between Demographic and Clinical Characteristics of Patients and Causal Attributions

The associations between causal attribution differences and demographic and clinical characteristics were examined (Table 3). Patients who were currently employed were significantly more likely to believe that an environmental factor was a cause of breast cancer compared to unemployed patients (*p* = 0.012). Patients with a family history of cancer were more likely to believe that personality and genetics/family history were causes of breast cancer compared to patients without a family history of cancer (*p* = 0.029, *p* = 0.007, respectively). In particular, patients with a family history of breast cancer were more likely to believe that genetics/family history was a cause of breast cancer compared to patients without a family history of breast cancer (*p* < 0.001). Compared to non-menopausal patients, menopausal patients were more likely to believe that a lack of prevention was a cause of breast cancer (*p* = 0.030). No significant differences were found between causal attributions and age, education level, marital status, cancer stage, type of surgery, chemotherapy, or radiation therapy of patients.

### 3.4. Relationships between Causal Attributions

There were significant differences in the three causal attributions according to family history of cancer in univariate analysis. We explored the relationships between causal attributions within the two groups (having no family history of cancer and having a family history of cancer).

**Figure 3:**
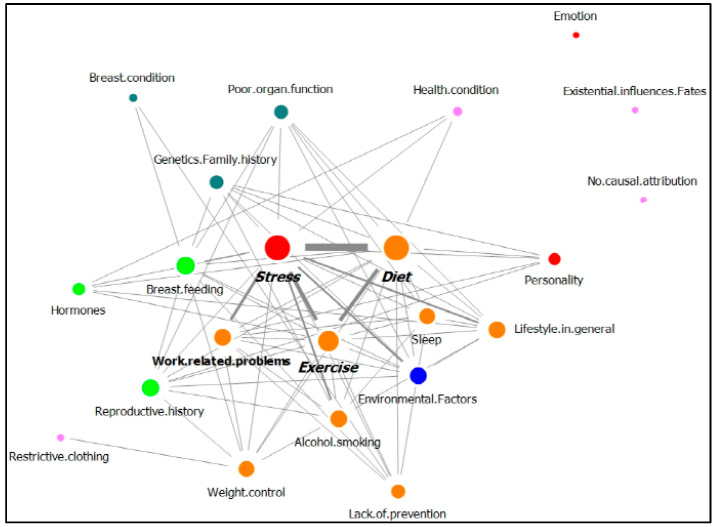
Network of causal attributions of breast cancer in patients with no family history of cancer (nodes of the same color mean that they belong to the same category.).

**Figure 4:**
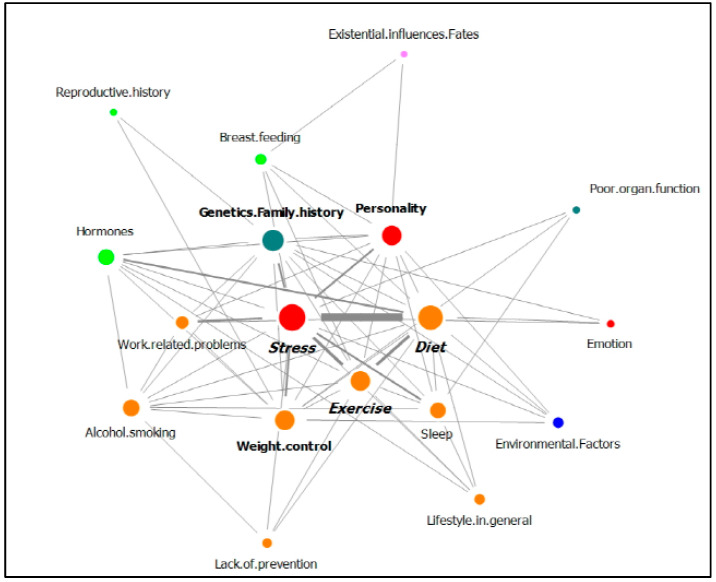
Network of causal attributions of breast cancer in patients with a family history of cancer (nodes of the same color mean that they belong to the same category.).

Table 4 shows the network of the top 10 nodes among the 23 nodes that represent breast cancer causal attributions, which were provided by breast cancer patients. Table 4 includes the frequency and the degree of the nodes identified in the replies of patients with no family history of cancer and with a family history of cancer. Figure 3 and Figure 4 display the network of the relationships between the total nodes, including the degree and the edge weight of the nodes identified in the answers from patents with no family history of cancer (Figure 3) and with a family history of cancer (Figure 4).

For patients with no family history of cancer, stress was the most frequent answer, followed by diet and exercise. These three nodes were connected to more than 10 nodes. As shown in Figure 3, these three nodes had the largest node size (degree), and they were connected to each other by a thick edge due to the high frequency of co-occurrence. Work-related problems and alcohol/smoking were also strongly connected with stress.

In the replies from patients with a family history of cancer, stress was the most frequent, followed by diet and exercise. Stress, diet, and exercise were connected to more than 10 nodes, as were genetics/family history, personality, and weight control. Figure 4 shows that six nodes had a large degree, and stress and the other five nodes were connected by a thick edge due to their high frequency of co-occurrence.

## 4. Discussion

In this study, we explored the causal attributions of breast cancer in Korean patients with breast cancer who were planning to undergo AET.

Overall, the most common causal attribution was stress, appearing for 72.6% of all respondents. This finding was consistent with those of previous studies that explored causal attributions among breast cancer patients. A previous study conducted with Korean breast cancer patients found that “stress and worry” were believed to be the most likely causes of breast cancer [22]. In other studies, conducted with Australian and Japanese women with breast cancer, it was found that most of the women agreed that stress (61.5%, 70%) was implicated in their own breast cancer [21,29]. On the contrary, in a study with Jewish Israeli women with breast cancer, more than half of the respondents answered that the cause of the disease was fate (70.0%) or God’s will (60.6%) [30]. Health beliefs can be influenced by patient experiences and culture [16]. In a qualitative study that explored causal attributions among Chinese American, Korean American, and Mexican American breast cancer survivors, stress was most often referenced; however, Korean American women tended to highlight work and lifestyle-related stress, while Mexican American women referenced family stress as contributing to their cancer, and Chinese American women tended to be less specific about the types of stress [16]. We need to provide tailored care for patients with breast cancer that considers individual experiences and cultural differences.

For a long time, many women have believed that stress causes breast cancer; however, epidemiological studies have not revealed a consistent association between stress and the development of breast cancer. A systematic review [31] identified 26 articles that supported and 18 articles that were not supportive of a link between stress and the incidence of breast cancer. Meanwhile, in several meta-analyses [32,33], it was concluded that high-intensity stress, such as the death of a spouse, could not be ruled out as a risk factor for breast cancer, although stressful life events are not generally associated with the risk of breast cancer in women. A few plausible explanations based on experimental studies of the potential relationship between high-intensity stress and the development of breast cancer are that stress can disturb many areas of the immune system, and impaired immune system function predisposes tissues to malignant growth [34]. In addition, it was suggested in an animal model that stress enhances glucocorticoid synthesis and glucocorticoid receptors in breast tissue and can enhance mammary tumor growth [35]. However, the role of glucocorticoids in human breast tissue can be complex and unpredictable [36,37]. Therefore, exposure to stressful life events, combined with the prolonged or elevated presence of cortisol, can increase the risk of breast cancer [38]. Stress might be considered an uncontrollable factor by breast cancer patients [26], and it might generate negative feelings, such as guilt, sadness, and anger, thus damaging the emotional adjustment of these women and their ability to effectively cope with the illness [12]. In contrast, other studies have reported that breast cancer patients who perceived stress as the main cause of breast cancer were more likely to engage in stress-reducing activities, such as yoga and meditation [39], and avoid stressful situations [40], and suggested that these activities may have a positive health benefit.

In this study, personality, one of the psychological factors, was found to be perceived as the cause of breast cancer by about 8% of patients. The personality types that patients mentioned were, for example, anxious, sensitive, meticulous, introverted, and emotionally suppressive personalities. Although personality is not considered a risk factor for breast cancer by experts [34,41,42,43], personality traits could affect the psychological adjustment of breast cancer patients [44]. The personality types mentioned above by the patients in this study are psychologically stressful attitudes and could have a negative effect on cancer prognosis. Therefore, healthcare providers should provide information about stress-reducing activities after stress detection and an assessment of personality traits.

Lifestyle factors, such as diet, lack of exercise, work-related problems, obesity, lack of sleep, and alcohol consumption/smoking were identified as the second to seventh highest breast cancer causal attributions. Almost half of the patients perceived diet as the cause of breast cancer, and more than 20% of the patients believed a lack of exercise was the causal reason for their breast cancer. This finding was similar to those of previous studies. Ferrucci et al. [17] found that lifestyle was reported by 60.8% of participants as the leading cause of their breast cancer. In a study by Lee et al., it was also reported that the second, third, and fifth most frequently mentioned causal attributions of breast cancer were behavioral factors, such as diet, poor medical care in the past, and overwork [22]. There is convincing scientific evidence that lifestyle factors, such as physical inactivity, a high-fat diet, weight gain, and the consumption of alcoholic drinks, are associated with an increased risk for breast cancer [45]. Patients who consider their lifestyle-related factors to be causes of breast cancer are more likely to make efforts to improve their lifestyle [16,20], and lifestyle modification has positive effects on breast cancer outcomes. Therefore, healthcare providers should educate patients about various interventions to improve their lifestyle behaviors.

Biological factors, such as genetics, hormones, and reproductive history, were identified as a causal attribution in about 4–7% of respondents. Biological factors are important causes of breast cancer, but this study found that very few breast cancer patients perceived it that way. Although the frequency of attribution to biological factors was low, patients with a family history of cancer answered that genetics was a causal attribution at a higher frequency (11.5%) than those without a family history of cancer (3.6%). In particular, genetic factors were provided as an answer by 31.3% of patients with a family history of breast cancer. We know that patients with a family history of cancer recognize family history as an important cause of breast cancer. On the other hand, it is known that genetic factors play an important role in the development of breast cancer in women who are diagnosed with breast cancer under the age of 40 years [46]. However, in this study, the frequency of the attribution of genetic factors as the cause of breast cancer was no different among women under 40 years old (6.9%) and older than 40 years (6.6%). Considering that young age is one of the risk factors for hereditary breast cancer, it is not clear why younger breast cancer patients do not refer to genetic factors more as the cause of breast cancer. This may be the result of deliberately trying to recognize modifiable and controllable risk factors rather than genetic factors. Another reason may be a lack of knowledge about hereditary breast cancer. Health care providers should provide education and counseling to build positive awareness of breast cancer, even if genetic factors are non-modifiable and uncontrollable risk factors. In addition, education and genetic counseling on hereditary breast cancer should be provided for high-risk groups.

We analyzed the relationships between patient characteristics and causal attributions of breast cancer. No significant differences were found between the causal attributions and the age, education level, marital status, treatment history, or cancer stage of patients. However, there were statistical differences among the causal attributions of personality, genetics, and reproductive history according to the patient’s family history of cancer. Patients with a family history of cancer were more likely to believe that genetics or personality was the cause of their cancer, along with stress. Having a family history of cancer is an important factor in breast cancer development [47]. It was reported that women with one or more first-degree relatives affected with breast cancer had a 1.37–2.45 times higher risk of breast cancer, compared with women with no affected first-degree relatives [48]. People with a family history of cancer may know more about cancer information and become more aware of their cancer risk, which may result in more frequent screening for the early detection of cancer [49]. It is unclear why personality was more frequently thought to be a cause of breast cancer along with stress in patients with a family history of cancer. However, it is supposed that they may have persistent anxiety and concerns that they could get cancer as they go through their family member’s cancer diagnosis and treatment process, and that their personality may have changed into a personality that did not express feelings or worries due to caring for family members who were cancer patients. They might believe that those personalities cause breast cancer. As seen in previous research, personality has not been identified as a risk factor for breast cancer. It is necessary to provide the correct information to breast cancer patients that personality, which cannot be changed easily, is not a risk factor for developing breast cancer.

This study has several limitations. First, as the perception of the cause of breast cancer was analyzed using data from patients in one hospital, the generalizability of the study findings is limited. However, patient age and clinical characteristics (e.g., stage) in this study are typical of those of breast cancer patients in Korea [50], indicating a certain level of representativeness of the study population. Second, stress, which was most frequently mentioned as a causal attribution, may have various causes, which may be an important concern for stress control; however, reasons for stress were not identified in this study. In addition, there was a limit to understanding the reasons why certain patients were more likely to perceive a specific cause of breast cancer. A deeper understanding of causal attributions can be explored through further interviews with patients. Third, future longitudinal study using structural equation models is needed to determine whether the causal attributions perceived by patients lead to changes in behavior that affect breast cancer progression and prognosis.

## 5. Conclusions

We explored causal attributions among Korean breast cancer patients planning to undergo AET as well as the relationships between these causal attributions and patient demographic and clinical characteristics. We found that the most frequently mentioned causal attribution was stress, followed by lifestyle-related factors, such as diet, exercise, and weight control. There has not been a consistent association between stress and the development of breast cancer in previous studies; however, it can have positive and negative effects on coping with breast cancer. Moreover, the perception of lifestyle factors as the cause of breast cancer can also lead to positive efforts to make lifestyle improvements. Thus, it is necessary to implement interventions to reduce stress and manage lifestyles that have positive effects on adherence to AET. Additionally, patients with a family history of cancer are more likely to believe that genetic factors and personality, as well as stress, are the causes of breast cancer. Thus, patients with a family history of cancer should be informed that there is no association between the incidence of breast cancer and personality, which is considered to be a non-modifiable factor. The implications of this study will allow healthcare providers to deliver more efficient care and education to breast cancer patients.

## Figures and Tables

**Table 1:** Categories and subcategories of causes attributed to breast cancer.

Categories	Subcategories
Biological factors	Genetics/Family history
	Poor organ function
	Breast condition
Environmental factors	Environmental factors
Behavioral factors	Lifestyle in general
	Exercise
	Diet
	Weight control
	Alcohol/Smoking
	Sleep
	Lack of prevention
	Work-related problems
Reproduction-related factors	Reproductive history
	Breastfeeding
	Hormones
Psychological factors	Emotion
	Personality
	Stress
Others	Low income
	Existential influences/Fates
	Health condition
	Restrictive clothing
	No causal attribution

**Table 2:** Patient demographic and clinical characteristics (*n* = 299).

Characteristics		Mean (SD ^1^) or n (%)
Age (years)		46.75 (8.18)
	≤40	58 (19.4)
	>40	241 (80.6)
Education level	High School or below	114 (38.1)
	College and above	185 (61.9)
Marital status	No	40 (13.4)
	Yes	257 (86.0)
	No information	2 (0.7)
Employment status	No	146 (48.8)
	Yes	153 (51.2)
Family history of cancer	No	169 (56.5)
	Yes	130 (43.5)
Family history of breast cancer	No	267 (89.3)
	Yes	32 (10.7)
Cancer stage	In situ	39 (13.0)
	Invasive (Stage I, II, or III)	260 (87.0)
Type of surgery	Mastectomy	57 (19.1)
	Conservation	242 (80.9)
Chemotherapy	No	199 (66.6)
	Yes	100 (33.4)
Radiation therapy	No	51 (17.1)
	Yes	248 (82.9)
Menopausal status	No	226 (75.6)
	Yes	73 (24.4)

^1^ SD: Standard Deviation.

**Table 3:** Causal attributions according to patient demographic and clinical characteristics (*n* = 299).

Causes	Age		Education Level		Marital Status		Employment Status		Family History of Cancer		Family History of Breast Cancer		Cancer Stage		Type of Surgery		Chemotherapy		Radiation Therapy		MenopausalStatus	
	≤40 (n = 58)	>40 (n = 241)	High school or below (n = 114)	College and above (n = 185)	No (n = 40)	Yes (n = 257)	No (n = 146)	Yes (n = 153)	No (n = 169)	Yes (n = 130)	No (n = 267)	No (n = 226)	Yes (n = 73)	Invasive (n = 260)	Mastectomy (n = 57)	Conservation (n = 242)	No (n = 199)	Yes (n = 100)	No (n = 51)	Yes (n = 248)	No (n = 226)	Yes (n = 73)
Stress (n = 217)	74.1	69.3	69.3	70.8	67.5	70.4	69.9	70.6	69.8	70.8	70.8	65.6	69.2	70.4	66.7	71.1	71.4	68.0	72.5	69.8	72.6	63.0
Diet (n = 138)	56.9	42.7	46.5	44.9	50.0	45.1	42.5	48.4	46.2	44.6	46.8	34.4	38.5	46.5	49.1	44.6	47.7	41.0	56.9	43.1	46.5	42.5
Exercise (n = 68)	15.5	24.5	23.7	22.2	15.8	24.1	22.6	22.9	24.9	20.0	22.5	25.0	25.6	22.3	19.3	23.6	23.1	22.0	23.5	22.6	21.7	26.0
Work-related problems (n = 34)	8.6	12	9.6	12.4	12.5	10.9	8.2	14.4	13.0	9.2	12.0	6.3	7.7	11.9	8.8	12.0	9.0	16.0	7.8	12.1	10.6	13.7
Weight control (n = 29)	12.1	9.1	7.9	10.8	5.0	10.5	11.6	7.8	7.7	12.3	9.0	15.6	10.3	9.6	10.5	9.5	8.5	12.0	5.9	10.5	9.7	9.6
Sleep (n = 27)	13.8	7.9	7.0	10.3	17.5	7.8	8.9	9.2	6.5	12.3	9.0	9.4	7.7	9.2	14.0	7.9	8.5	10.0	9.8	8.9	10.6	4.1
Alcohol/Smoking (n = 26)	6.9	9.1	9.6	8.1	15.0	7.0	11.0	6.5	9.5	7.7	9.4	3.1	5.1	9.2	7.0	9.1	6.5	13.0	2.0	10.1	9.3	6.8
Personality (n = 25)	3.4	8.7	7.0	8.1	2.5	8.6	7.5	7.8	4.7 *	11.5 *	6.7	15.6	10.3	7.3	7.0	7.9	8.0	7.0	3.9	8.5	7.1	9.6
Genetics/Family history (n = 21)	6.9	6.6	4.4	8.6	5.0	7.4	7.5	6.5	3.6 **	11.5 **	4.1 ***	31.3 ***	7.7	6.9	7.0	7.0	6.0	9.0	5.9	7.3	8.4	2.7
Environmental Factors (n = 20)	3.4	7.1	5.3	7.0	2.5	7.0	2.7 *	9.8 *	7.7	4.6	6.4	6.3	7.7	6.2	5.3	6.6	7.5	4.0	5.9	6.5	6.2	6.8
Lifestyle in general (n = 20)	6.9	6.6	9.6	4.9	12.5	5.8	5.5	7.8	8.3	4.6	7.1	3.1	5.1	6.9	7.0	6.6	6.0	8.0	3.9	7.3	7.1	5.5
Hormones (n = 18)	5.2	6.2	3.5	7.6	7.5	5.8	7.5	4.6	4.1	8.5	6.4	3.1	12.8	5.0	3.5	6.6	7.5	3.0	2.0	6.9	5.3	8.2
Lack of prevention (n = 17)	5.2	5.4	5.3	5.4	2.5	5.8	5.5	5.2	5.9	4.6	6.0	0.0	5.1	5.4	5.3	5.4	6.5	3.0	5.9	5.2	3.5 *	11.0 *
Breastfeeding (n = 16)	5.2	5.4	2.6	7.0	0.0	6.2	4.8	5.9	7.1	3.1	5.6	3.1	5.1	5.4	5.3	5.4	5.0	6.0	2.0	6.0	6.2	2.7
Reproductive history (n = 14)	3.4	4.6	2.6	5.4	10.0	3.5	2.7	5.9	7.1 **	0.8 **	4.5	3.1	2.6	4.6	0.0	5.4	5.0	3.0	2.0	4.8	4.0	5.5
Poor organ function (n = 7)	1.7	2.5	1.8	2.7	0.0	2.7	2.7	2.0	3.0	1.5	2.6	0.0	2.6	2.3	1.8	2.5	3.0	1.0	2.0	2.4	2.2	2.7
Emotion (n = 3)	0	1.2	0.9	1.1	0.0	1.2	2.1	0.0	0.0	2.3	0.7	3.1	0.0	1.2	3.5	0.4	1.5	0.0	2.0	0.8	0.4	2.7
Existential influences/Fates (n = 2)	0	0.8	0.9	0.5	0.0	0.8	0.0	1.3	0.6	0.8	0.7	0.0	0.0	0.8	0.0	0.8	1.0	0.0	0.0	0.8	0.4	1.4
Health condition (n = 2)	0	0.8	0.9	0.5	0.0	0.8	0.7	0.7	1.2	0.0	0.7	0.0	0.0	0.8	0.0	0.8	1.0	0.0	0.0	0.8	0.9	0.0
Breast condition (n = 1)	1.7	0	0.0	0.5	0.0	0.4	0.7	0.0	0.6	0.0	0.4	0.0	0.0	0.4	1.8	0.0	0.0	1.0	0.0	0.4	0.4	0.0
Restrictive clothing (n = 1)	1.7	0	0.0	0.5	0.0	0.4	0.0	0.7	0.6	0.0	0.4	0.0	0.0	0.4	0.0	0.4	0.0	1.0	0.0	0.4	0.4	0.0
No causal attribution (n = 1)	0	0.4	0.0	0.5	0.0	0.4	0.7	0.0	0.6	0.0	0.4	0.0	0.0	0.4	0.0	0.4	0.0	1.0	0.0	0.4	0.0	1.4

*** *p* < 0.001, ** *p* < 0.01, * *p* < 0.05; Numbers in bold type mean that the *p*-value is less than 0.05.

**Table 4:** The nodes with the frequency and degree of patients with no family history of cancer and a family history of cancer.

No Family History of Cancer (*n* = 169)				Family History of Cancer (*n* = 130)			
Causes	Frequency	Causes	Degree	Causes	Frequency	Causes	Degree
Stress	120	Diet	16	Stress	97	Stress	15
Diet	80	Stress	16	Diet	58	Diet	14
Exercise	42	Exercise	12	Exercise	26	Genetics/Family history	12
Work-related problems	22	Breastfeeding	10	Personality	17	Exercise	11
Alcohol/Smoking	16	Environmental Factors	9	Weight control	16	Personality	11
Environmental Factors	14	Lifestyle in general	9	Sleep	16	Weight control	11
Lifestyle in general	14	Alcohol/Smoking	9	Genetics/Family history	15	Alcohol/Smoking	9
Weight control	13	Work-related problems	9	Work-related problems	12	Sleep	8
Reproductive history	13	Reproductive history	9	Hormones	11	Hormones	8
Breastfeeding	12	Weight control	8	Alcohol/Smoking	10	Work-related problems	6

## Data Availability

The datasets used and/or analyzed during the current study are available from the corresponding author on reasonable request.
